# Splice-Site Mutations Cause Rrp6-Mediated Nuclear Retention of the Unspliced RNAs and Transcriptional Down-Regulation of the Splicing-Defective Genes

**DOI:** 10.1371/journal.pone.0011540

**Published:** 2010-07-12

**Authors:** Andrea B. Eberle, Viktoria Hessle, Roger Helbig, Widad Dantoft, Niclas Gimber, Neus Visa

**Affiliations:** Department of Molecular Biology and Functional Genomics, Stockholm University, Stockholm, Sweden; Emory University, United States of America

## Abstract

**Background:**

Eukaryotic cells have developed surveillance mechanisms to prevent the expression of aberrant transcripts. An early surveillance checkpoint acts at the transcription site and prevents the release of mRNAs that carry processing defects. The exosome subunit Rrp6 is required for this checkpoint in *Saccharomyces cerevisiae*, but it is not known whether Rrp6 also plays a role in mRNA surveillance in higher eukaryotes.

**Methodology/Principal Findings:**

We have developed an *in vivo* system to study nuclear mRNA surveillance in *Drosophila melanogaster*. We have produced S2 cells that express a human β-globin gene with mutated splice sites in intron 2 (*mut* β-globin). The transcripts encoded by the *mut* β-globin gene are normally spliced at intron 1 but retain intron 2. The levels of the *mut* β-globin transcripts are much lower than those of wild type (*wt*) ß-globin mRNAs transcribed from the same promoter. We have compared the expression of the *mut* and *wt* β-globin genes to investigate the mechanisms that down-regulate the production of defective mRNAs. Both *wt* and *mut* β-globin transcripts are processed at the 3′, but the *mut* β-globin transcripts are less efficiently cleaved than the *wt* transcripts. Moreover, the *mut* β-globin transcripts are less efficiently released from the transcription site, as shown by FISH, and this defect is restored by depletion of Rrp6 by RNAi. Furthermore, transcription of the *mut* β-globin gene is significantly impaired as revealed by ChIP experiments that measure the association of the RNA polymerase II with the transcribed genes. We have also shown that the *mut* β-globin gene shows reduced levels of H3K4me3.

**Conclusions/Significance:**

Our results show that there are at least two surveillance responses that operate cotranscriptionally in insect cells and probably in all metazoans. One response requires Rrp6 and results in the inefficient release of defective mRNAs from the transcription site. The other response acts at the transcription level and reduces the synthesis of the defective transcripts through a mechanism that involves histone modifications.

## Introduction

In eukaryotes, precursor mRNA molecules (pre-mRNAs) are synthesized by RNA polymerase II (Pol-II) and processed in the nucleus into mature mRNAs. Processing of almost all protein-coding pre-mRNAs comprises capping of the 5′end, intron removal, cleavage and polyadenylation of the 3′end. The mature mRNAs are exported through the nuclear pore complex to their site of translation in the cytoplasm. The transcripts are associated with different types of RNA-binding proteins throughout the gene expression pathway, forming ribonucleoprotein complexes (RNPs) [reviewed in 1], [ 2].

The individual steps of gene expression can be reproduced *in vitro* independently of each other. In the cell, however, these reactions are often coordinated and influence each other in several ways [3]. Pre-mRNA processing often takes place cotranscriptionally [4], and the processing and transcription machineries interact with each other at the gene. The binding of pre-mRNA processing factors to the transcription machinery and to chromatin remodeling enzymes couples the pre-mRNA processing to transcription and makes the processing reactions more efficient [5]. In some cases, the interactions serve regulatory purposes. For instance, the rates of transcription elongation affect splice site selection and therefore affect the outcome of alternative splicing [6]. Splicing can also affect transcription. Spliceosomal UsnRNPs interact with transcription elongation factors in human cells and stimulate transcription elongation through a mechanism that requires the presence of splicing signals in the pre-mRNA [7]. *In vivo* experiments based on the use of integrated transgenes expressing wild-type mRNAs or mRNAs carrying a 5′splice-site mutation in a promoter-proximal intron revealed a strong correlation between splicing efficiency and transcriptional activity [8]. Interactions between the pre-mRNA processing and transcription machineries also provide checkpoints for the quality control of mRNA biogenesis [reviewed in 9]–[11].

Recent studies have shown that transcripts with structural defects, such as premature translation termination codons or retained introns, are relatively abundant *in vivo* [12], [13]. Defective transcripts can originate from mutations in the genome, from defects in RNP assembly or from pre-mRNA maturation errors. Since accurate gene expression is an essential requirement for all living cells, several surveillance mechanisms have evolved at the RNA level in both the nucleus and the cytoplasm [reviewed in 14]–[16]. These mechanisms ensure that defective transcripts are identified and eliminated, and thus prevent the production of erroneous and potentially harmful proteins. The exosome, a multiprotein complex with ribonuclease activity, plays a crucial role in the recognition and degradation of defective transcripts. The eukaryotic exosome consists of a catalytically inactive nine-subunit core that associates with the ribonucleases Rrp6 and/or Dis3/Rrp44 [reviewed in 17], [ 18]. The exosome has many functions in RNA biology in addition to its role in RNA surveillance, and is involved in the processing of non-coding RNA precursors, the turnover of mRNA and the degradation of cryptic unstable transcripts. This means that the specificity and regulation of the multiple activities of the exosome require specific co-factors [reviewed in 19]–[21]. Studies in *Drosophila melanogaster* (*D. melanogaster*) have revealed that the exosome is recruited cotranscriptionally to active genes through interactions with the transcription machinery [22] and with hnRNP proteins that bind to the nascent pre-mRNA [23].

In budding yeast and mammalian cells, unprocessed pre-mRNAs are not exported efficiently to the cytoplasm, and accumulate at the transcription site [24]–[26]. Studies in *Saccharomyces cerevisiae* have shown that this cotranscriptional surveillance mechanism depends on the protein Rrp6 and the exosome [25] . The final maturation and release of export-competent mRNPs from the transcription site requires in mammalian cells the carboxy terminal domain (CTD) of the large subunit of Pol-II [27]. However, it is not known whether Rrp6 and the exosome play any role in this process or in the nuclear retention of defective transcripts in metazoans.

We have developed a system to study the expression of splicing-defective transcripts, in order to obtain further insight into the links between cotranscriptional splicing and surveillance in metazoans. We have produced *D. melanogaster* S2 cells that express either a wild-type (*wt*) human β-globin gene or a *mut*ated version (*mut*) that is not spliced properly. We have analyzed the transcription of the *wt* and *mut* β-globin genes, the processing of the *wt* and *mut* β-globin transcripts, and the release of the transcripts from the transcription site under normal conditions and after depletion of Rrp6 by RNA interference (RNAi). Our results show that at least two different types of splicing surveillance operate at the transcription site and that the role of Rrp6 in cotranscriptional surveillance of mRNA biogenesis is conserved from yeast to metazoans.

## Results

### A *Drosophila* cell system to study RNA retention at the transcription site

**Figure 1:**
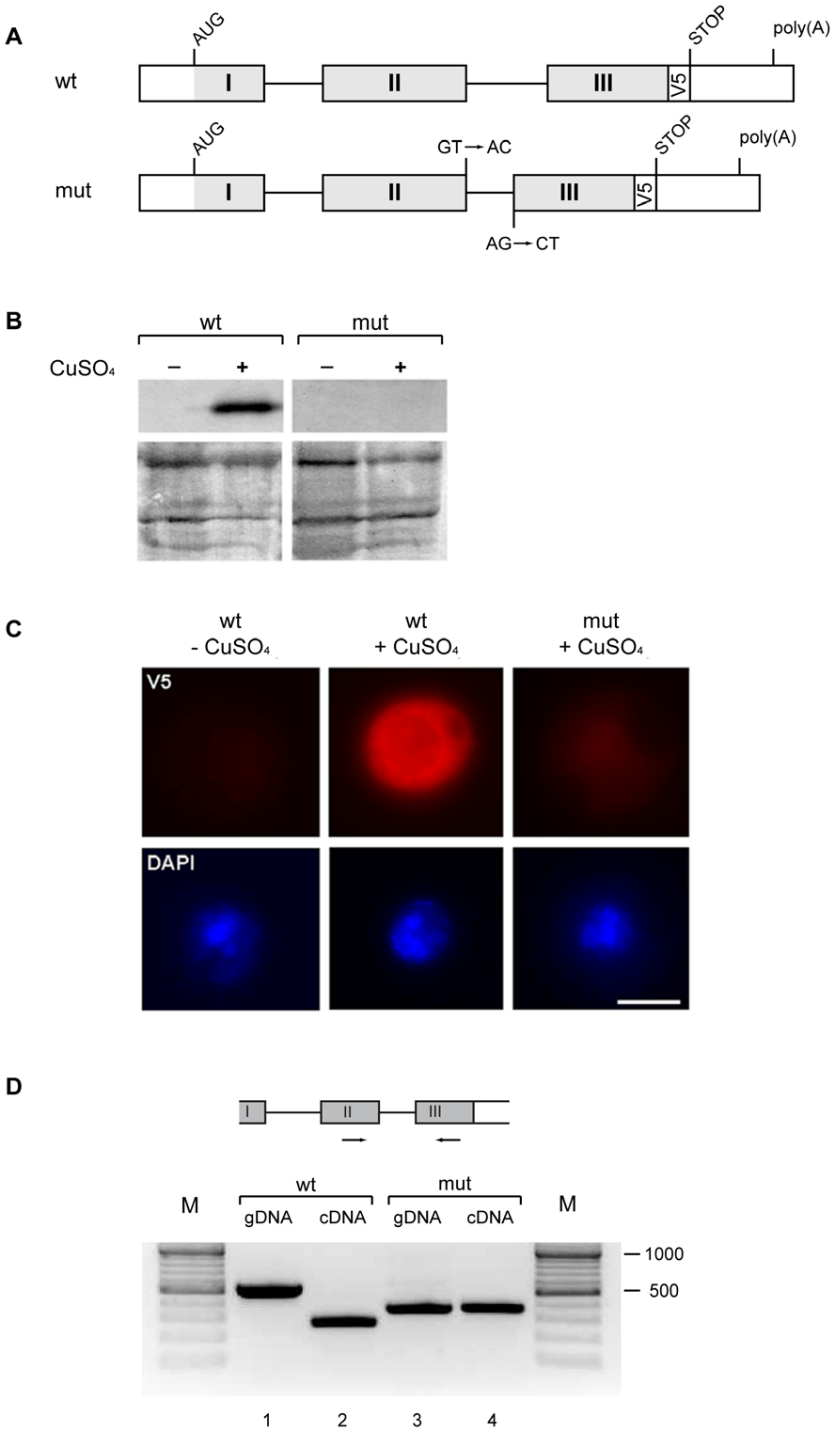
The human β-globin genes expressed in *Drosophila* S2 cells. (**A**) Schematic representation of the β-globin transcripts expressed in S2 cells. The grey boxes represent the coding β-globin sequences. The white boxes indicate the 5′ UTR and 3′ UTR of the pMT vector (see Materials and Methods S1 for details). The box marked *V5* indicates the position of the V5 tag. The *mut* RNA carries a shorter intron 2 and mutations in both the 5′ and 3′ splice sites of intron 2, as indicated in the figure. (**B**) Western blot analysis of the expression of the *wt* and *mut* β-globin genes. The expression of the β-globin genes was induced with 500 µM CuSO_4_ for 24 h and analyzed by Western blotting using the anti-V5 antibody. Protein expression was detected only from the *wt* construct. As a loading reference, a section of the PVDF filter containing proteins in the 50–90 kDa range was stained for total protein with Coomassie blue. (**C**) The expression of the β-globin genes analyzed by immunofluorescence. Expression of the *wt* and *mut* β-globin genes was induced as described above and the cells were stained with the anti-V5 antibody (*red*) to visualize β-globin expression. DAPI counterstaining was used to visualize the nuclei (*blue*). Exposure times were the same for all images. The magnification bar represents 5 µm. (**D**) The β-globin transcripts analyzed by RT-PCR. The expression of the *wt* and *mut* β-globin genes was induced as described above. Total RNA was purified and reverse-transcribed, and the β-globin sequences were amplified by PCR primers flanking intron 2, as indicated in the figure (*lanes 2* and *4*). The genomic DNA isolated from β-globin *wt* or *mut* S2 cells was used in parallel to check the splicing pattern (gDNA, *lanes 1* and *3*). Molecular mass standards are shown (*M*) and the length of the major bands is indicated in bp.

Wild-type (*wt*) and mutant (*mut*) versions of the human β-globin gene have been used in previous studies as reporter genes to analyze the surveillance mechanisms that operate during transcription. These versions of the gene have been used, in particular, to study the retention of unprocessed pre-mRNAs at the transcription site in mammalian cells [24], [27]. We have applied the same approach to set up a *Drosophila* cell system to study nuclear retention of unspliced transcripts. For this purpose, we subcloned the *wt* β-globin gene and a *mut* version of it into a vector that was suitable for expression in *Drosophila melanogaster* S2 cells (see Materials and Methods S1). The *wt* and the *mut* sequences were tagged with a V5 epitope to facilitate expression analyses. The *mut* RNA carries a GT to AC mutation in the 5′ splice site and an AG to CT mutation in the 3′ splice site of the second intron (Figure 1A). These mutations impair removal of the second intron and, in human cells, cause retention of the RNA at the transcription site [24].

Plasmids carrying the *wt* and *mut* β-globin genes under the control of an inducible metallothionein promoter were transfected into S2 cells, and stable transfectants were selected. Western blot analysis and immunofluorescent staining with an anti-V5 antibody indicated that the *wt* construct was expressed, as expected. In contrast, no protein product could be detected in cells that expressed the *mut* β-globin gene (Figures 1B and 1C). However, the *mut* RNA could be detected by RT-PCR, which indicated that the *mut* β-globin gene was transcribed (Figure 1D). The primers used for the RT-PCR reaction shown in Figure 1D were complementary to sequences flanking intron 2. The same primers were used on genomic DNA (gDNA) to assess intron removal. PCR and RT-PCR reactions were also run with primers designed to amplify the entire β-globin gene, and all the amplicons were purified and sequenced (data not shown). Analysis of the sequences confirmed that intron 1 was correctly spliced in both *wt* and *mut* β-globin transcripts, whereas intron 2 was correctly spliced in the *wt* β-globin mRNAs but was retained in the *mut* transcripts, as expected (Figure S1).

### The *mut* β-globin genes are not efficiently released from the transcription site

**Figure 2:**
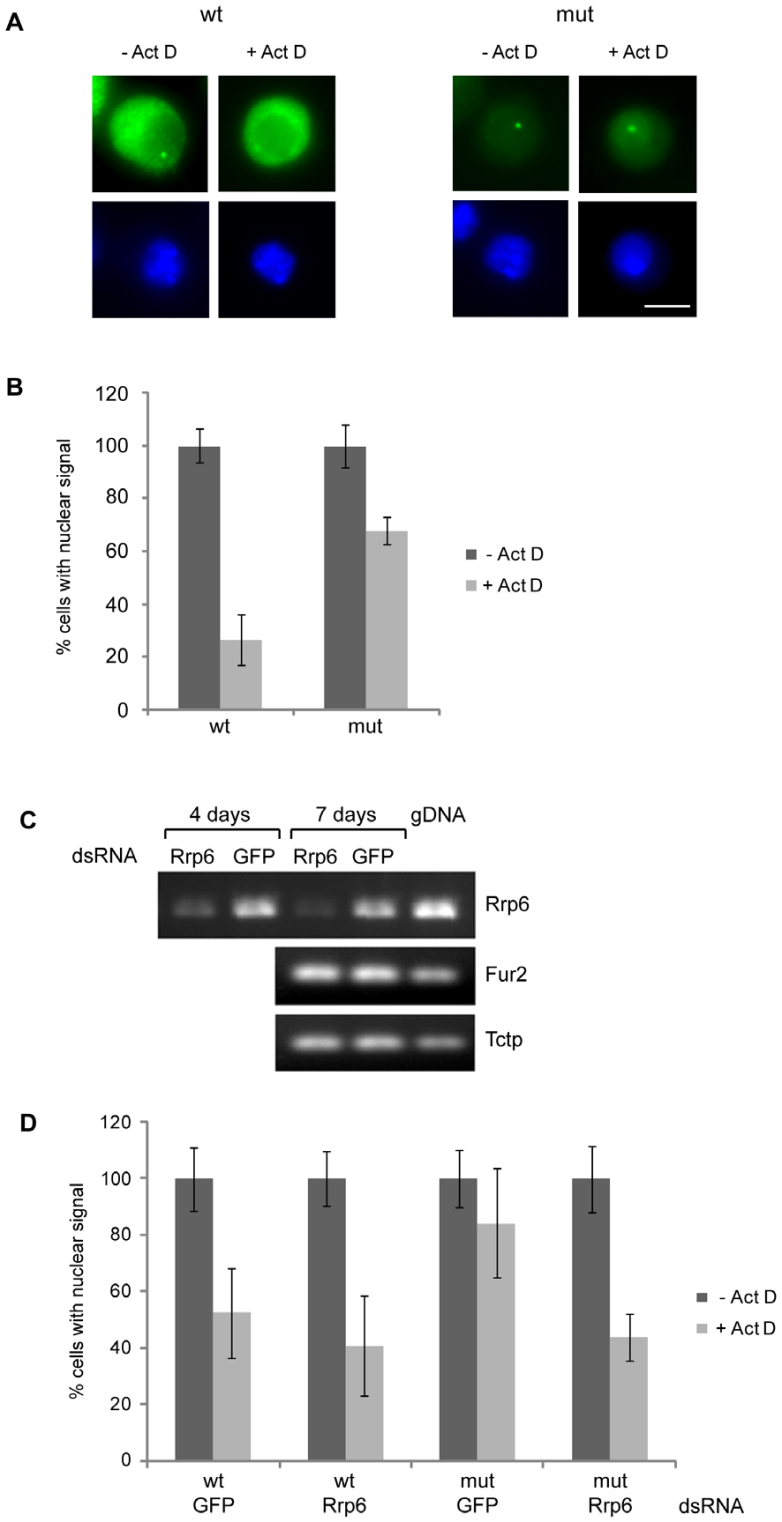
Nuclear retention of *mut*ant β-globin transcripts in S2 cells. (**A**) The location of the β-globin RNAs studied by FISH. Expression of the *wt* and *mut* β-globin genes was induced with 400 µM CuSO_4_ for 24 h and the location of the β-globin transcripts was analyzed by FISH. The β-globin sequence of the pβΔRS plasmid was labeled with digoxigenin and used as a probe (*green*). The preparations were counterstained with DAPI (*blue*). When indicated, the cells were treated with actinomycin D before fixation to study the presence of transcripts at the transcription site in the absence of ongoing RNA synthesis. The magnification bar represents 5 µm. (**B**) Quantitative analysis of the FISH experiments. The frequency of cells with an intensely fluorescent spot in the nucleus was counted in control cells (*Act-, dark bars*) and in cells treated with actinomycin D (*Act+, light bars*). For each treatment, 98 cells were analyzed. The experiment was carried out three independent times, with a total of 294 cells analyzed. To show the changes induced by actinomycin D, the results are expressed as average percentage relative to the frequencies obtained in non-treated cells. The error bars represent standard deviations of the mean (n = 3). Comparisons of *wt* to *mut* treated with actinomycin D using a paired, one-tailed Student's t-test gave *p* = 0.02, n = 3. (**C**) Depletion of Rrp6 by RNAi in S2 cells. S2 cells were treated with either Rrp6-dsRNA or control GFP-dsRNA. After 4 days or 7 days, as indicated, total RNA was purified and reverse-transcribed. The resulting cDNA was analyzed by PCR with primers specific for the *rrp6* gene. The expressions of two unrelated genes, *fur2* and *tctp*, were analyzed in parallel to assess the specificity of the treatment. Genomic DNA was analyzed in parallel. Equivalent amounts of cells expressing *wt* and *mut* transcripts were used for the analysis. (**D**) Depletion of Rrp6 restores the release of β-globin transcripts from the transcription site. The distribution of the β-globin transcripts was analyzed by FISH in cells treated with either Rrp6-dsRNA or GFP-dsRNA for 5 days. Induction, actinomycin treatment and FISH analysis were the same as those described for Figures 2A and 2B. The histogram shows the average proportion of cells with an intense fluorescent spot. *Dark bars* indicate data from non-treated control cells. *Light bars* correspond to cells treated with actinomycin D before fixation and FISH analysis. A total of 392 cells from two independent experiments, each in duplicate, was analyzed for each treatment. The error bars represent standard deviations (n = 4). Comparisons of cells expressing *mut* RNA and treated with Rrp6-dsRNA with those treated with GFP-dsRNA in the presence of actinomycin D using an unpaired, one-tailed Student's t-test gave *p* = 0.03, n = 4.

We applied fluorescent *in situ* hybridization (FISH) to determine whether *Drosophila* cells have a mechanism for the retention of unspliced mRNAs at the transcription site. For this purpose, we induced the expression of the *wt* and *mut* β-globin genes and analyzed the cellular distribution of the β-globin RNAs by FISH (Figure 2). Intense fluorescence was observed in the cytoplasm of cells that expressed the *wt* gene, which indicated that the *wt* mRNA was exported to the cytoplasm, as expected. Moreover, a bright fluorescent spot was observed in the nucleus in more than 60% of the cells (Figure 2A, left panel). Previous FISH experiments carried out in mammalian cells have shown that the bright fluorescent spot coincides with the β-globin gene and is due to the presence of newly synthesized transcripts that have not left the transcription site [24]. Custodio and coworkers showed that the frequency of cells showing a bright fluorescent spot is drastically reduced when transcription is inhibited [24]. The interpretation of this reduction is that, in the absence of ongoing transcription, the already synthesized transcripts leave the gene and the transcription site becomes depleted of RNA. We observed the same effect in the S2 cells that expressed the *wt* β-globin RNA. The frequency of cells with a nuclear spot was reduced by a factor of almost 4 when transcription was inhibited by actinomycin D (Figure 2B). This result is consistent with the observations in mammalian cells [24] and with the rapid release and efficient export of the *wt* mRNA.

The overall intensity of the FISH signal was much lower in cells that expressed the *mut* β-globin gene, and the fluorescence intensity in the cytoplasm was close to the background level. However, more than 40% of the cells displayed a bright fluorescent spot in the nucleus, similar to the one observed in cells that expressed the *wt* gene (Figure 2A, right panel). These observations agree with the results of the RT-PCR analyses (Figure 1D) and confirm that the *mut* β-globin gene is indeed transcribed. Treatment of the cells with actinomycin D resulted in a slight decrease in the frequency of cells with a bright nuclear spot. This decrease, however, was less pronounced than that of cells expressing the *wt* gene (Figure 2B). This difference suggests that the release of *mut* transcripts from the transcription site is not as efficient as the release of *wt* transcripts.

The low abundance of *mut* RNA in the cytoplasm and the high frequency of *mut* cells that show a nuclear FISH signal after transcription inhibition lead us to conclude that insect cells have a surveillance mechanism that inhibits the release of unprocessed RNAs from the transcription site.

### Rrp6 is required for retention of *mut* β-globin mRNAs at the transcription site

Rrp6 is needed for the nuclear retention of unprocessed transcripts in *S. cerevisiae* [reviewed in 9]. We hypothesized that the retention of unspliced RNAs at the transcription site requires Rrp6 also in metazoans. We tested this possibility using RNA interference (RNAi) to knock down the expression of Rrp6 in S2 cells that express either the *wt* or *mut* β-globin gene. The S2 cells were treated with dsRNA for Rrp6 or with a control dsRNA that was complementary to green fluorescent protein (GFP). The efficiency of the RNAi at different time points was analyzed by RT-PCR. Figure 2C shows that the Rrp6 mRNA was significantly down-regulated after 4 days of dsRNA treatment and was almost undetectable after 7 days. Moreover, the down-regulation was specific for Rrp6, as is shown by the fact that other unrelated mRNAs were not significantly affected by the RNAi treatment (see *fur2* and *tctp* in Figure 2C). The depletion of the Rrp6 protein was confirmed by Western blotting (Figure S2). Depletion of Rrp6 resulted in growth arrest (data not shown), which is consistent with a recent finding by Andrulis and coworkers, who showed that Rrp6 is important for cell growth and error-free mitosis [28]. Consequently, in order to minimize secondary effects due to growth perturbations, we chose to harvest the cells after 5 days of dsRNA treatment.

We knocked down the expression of Rrp6 by RNAi in the S2 cells that expressed either *wt* or *mut* β-globin genes, and analyzed the effect of the RNAi treatment on the release of the β-globin transcripts from the transcription site by FISH after treatment of the cells with actinomycin D. As in the experiments whose results are shown in Figure 2B, we counted the frequency of cells that showed a bright spot in the nucleus. In cells that expressed *wt* β-globin, depletion of Rrp6 did not have any significant effect on the retention of transcripts (Figure 2D). In cells that expressed *mut* β-globin in the absence of actinomycin D, there were no significant differences between the Rrp6-dsRNA samples and the GFP-dsRNA controls. However, in the presence of actinomycin D, the frequency of cells with a bright nuclear spot was significantly reduced by the Rrp6-dsRNA (*p* = 0.03 for cells expressing *mut* RNA and treated with Rrp6-dsRNA compared with those treated with GFP-dsRNA in the presence of actinomycin D). In summary, we conclude that Rrp6 is involved in the nuclear retention of the unspliced *mut* β-globin RNAs.

### The *mut* β-globin transcripts are cleaved and polyadenylated

We have investigated the mechanisms by which the unspliced *mut* mRNAs are retained at the transcription site by determining whether the 3′ end of the *mut* transcripts is normally processed. Typical Pol-II transcripts are processed at the 3′ end by a two-step reaction that includes an endonucleolytic cleavage of the nascent transcript downstream of the polyadenylation signal (pA) followed by addition of a poly(A) tail at the 3′ end of the upstream cleavage product [29]. The downstream product is unstable and is degraded by the 5′-3′ exoribonuclease Rat1 (Xrn2 in human) [30], [31].

**Figure 3:**
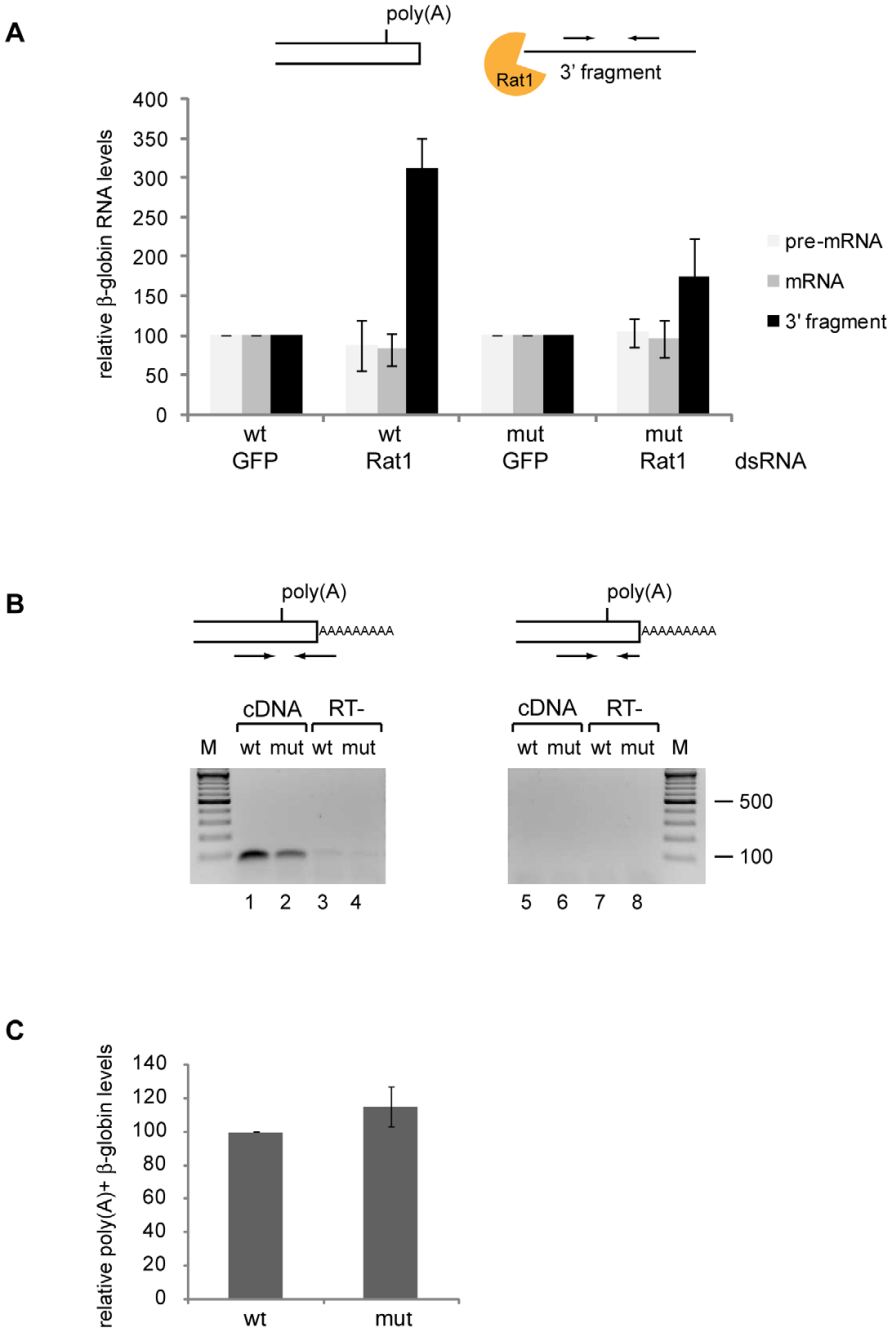
Cleavage and polyadenylation of the β-globin transcripts. (**A**) Detection of the downstream cleavage product (*3′ fragment*). The expression of Rat1 was silenced by RNAi in S2 cells expressing either *mut* or *wt* β-globin. Control cells were treated in parallel with GFP-dsRNA. Total RNA was purified and reverse-transcribed from dsRNA-treated cells, and the resulting cDNAs were quantified by qPCR with primers specific for the pre-mRNA, the mRNA (see Figure 4A) and the 3′ fragment (see the schematic picture above the histogram). The histogram shows the average values and standard deviations of β-globin levels, normalized to actin 5C RNA, from three independent experiments with two qPCR runs each (n = 6). The relative accumulation of downstream product was less pronounced in cells that expressed the *mut* β-globin gene than in cells that expressed the *wt* gene (*p*<0.01 using a paired, two-tailed Student's t-test, n = 6). (**B**) PCR-based detection of polyadenylated β-globin transcripts. Total RNA was purified from nuclear preparations of S2 cells expressing either *mut* or *wt* β-globin genes. The resulting cDNAs were used to amplify polyadenylated β-globin sequences by PCR with a 26 nt-long downstream primer complementary to the beginning of the poly(A) tail, as indicated in the figure (*lanes 1*–*4*). Control reactions with a 16 nt-long primer lacking the oligo(dT) extension were processed in parallel to rule out poly(A)-independent priming (*lanes 5*–*8*). Contamination with genomic DNA was assessed in parallel reactions without reverse transcriptase (*RT-*). (**C**) RT-qPCR quantification of polyadenylated β-globin transcripts. The samples described above were analyzed by RT-qPCR. Relative levels of polyadenylated β-globin transcripts, normalized to the total β-globin RNA, are shown. Values and standard deviations represent the average from two independent experiments with two qPCR runs each (n = 4). No significant differences were observed using a paired, two-tailed Student's t-test (*p* = 0.12, n = 4).

In a first series of experiments, we used RNA interference (RNAi) to knock down the expression of Rat1 in S2 cells that expressed either the *wt* or the *mut* β-globin genes, and we analyzed the levels of the downstream cleavage product by quantitative RT-PCR (RT-qPCR). The decreased expression of Rat1 was verified by RT-qPCR (Figure S3). The depletion of Rat1 increased the levels of the downstream product (*3′ fragment* in Figure 3A) for both *wt* and *mut* β-globin transcripts. However, the relative accumulation of downstream product was significantly less pronounced in cells that expressed the *mut* β-globin gene than in cells that expressed the *wt* gene (p<0.01). Altogether, these results indicated that both the *wt* and the *mut* β-globin transcripts are cleaved, but that the cleavage of the *mut* β-globin transcripts is less efficient.

We investigated in a second series of experiments whether the cleaved transcripts are polyadenylated. We designed a PCR-based experiment using a downstream primer complementary to the end of the β-globin 3′ UTR and the beginning of the poly(A) tail, as shown in Figure 3B. Total RNA was purified from nuclear preparations of S2 cells that expressed either the *wt* or the *mut* β-globin genes and this RNA was reverse-transcribed. RT-PCR products corresponding to polyadenylated transcripts were detected in both *wt* and *mut* β-globin cells (Figure 3B, left panel). Control RT-PCR reactions using a shorter primer that only overlaps with the 3′ UTR but not with the poly(A) tail failed to amplify the product to a level that could be detected, which indicates that the transcript-specific sequence alone is not sufficient to prime efficient PCR amplification in the conditions of our experiment (Figure 3B, right panel). We conclude that the PCR products that are detected originate from β-globin transcripts that are cleaved and polyadenylated. We quantified the abundance of the polyadenylated β-globin transcripts relative to the total quantity of β-globin RNAs by RT-qPCR, where the total β-globin RNAs had been amplified with primers complementary to exonic sequences. The level of polyadenylation of the *mut* β-globin transcripts was similar to that of *wt* (Figure 3C).

### Impaired transcription of the *mut* β-globin gene

**Figure 4:**
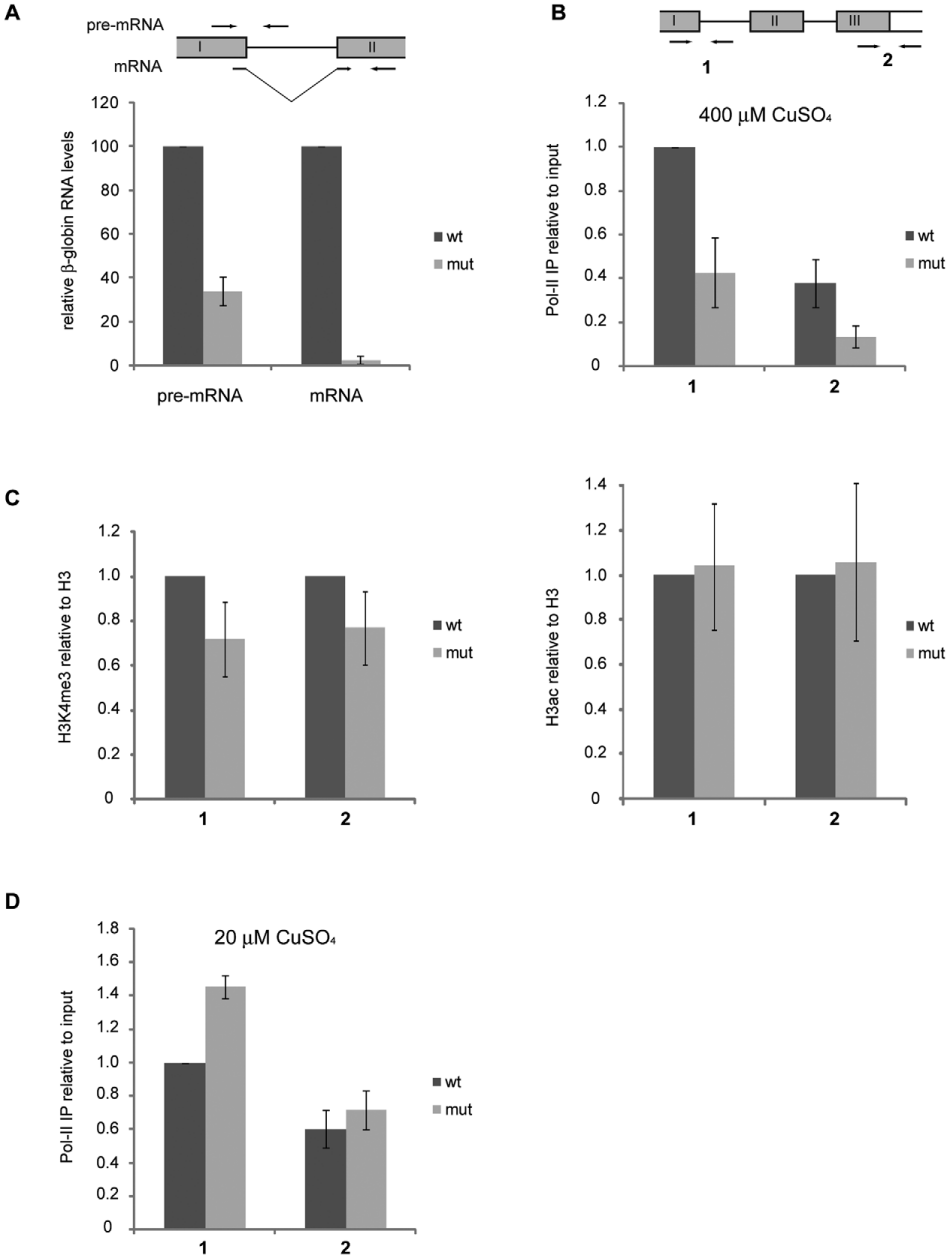
Expression of the *mut* and *wt* β-globin genes in S2 cells. (**A**) The relative levels of β-globin transcripts analyzed by RT-qPCR. The expression of the β-globin genes was induced with 400 µM CuSO_4,_ and total RNA was purified and reverse-transcribed. β-globin pre-mRNA and mRNA levels were quantified by RT-qPCR using specific primers as indicated in the figure. The histogram shows average relative transcript levels normalized to actin 5C. The error bars are standard deviations from three independent experiments, each in duplicate (n = 6). Comparisons between *wt* and *mut* using a paired, two-tailed Student's t-test gave *p*<0.001 and *p*<0.0001 (n = 6) for pre-mRNA and mRNA, respectively. (**B**) Analysis of Pol-II density in β-globin genes. Expression of the β-globin genes was induced as described above (400 µM CuSO_4_, high induction conditions) and the density of Pol-II in the *mut* and *wt* β-globin genes was analyzed by ChIP using an anti-CTD antibody. Two regions near the promoter (*region 1*) and the stop codon (*region 2*) of the β-globin gene were analyzed by qPCR using specific primers as indicated in the figure. The histogram shows average Pol-II signals relative to input and normalized to actin 5C from three independent experiments, each one quantified twice by qPCR (n = 6). The error bars represent standard deviations. Comparisons between *wt* and *mut* using a paired, two-tailed Student's t-test gave *p*<0.0001 and *p*<0.005 (n = 6) for regions 1 and 2, respectively. (**C**) Analysis of chromatin modifications in the β-globin genes (400 µM CuSO_4_, high induction conditions) by ChIP using antibodies against histone H3, H3K4me3 and H3ac. The immunoprecipitated DNA was measured by qPCR using primers for β-globin regions 1 and 2, as in (B). The histograms show the average signals for H3K4me3 (left) and H3ac (right) relative to histone H3 and normalized to actin 5C from three (H3K4me3) and two (H3ac) independent experiments, each quantified in duplicate (n = 6 and n = 4 for H3K4me3 and H3ac, respectively). The error bars represent standard deviations. Comparisons of H3K4me3 occupancy between *wt* and *mut* using a paired, two-tailed Student's t-test gave *p* = 0.06 and *p* = 0.04 (n = 6) for regions 1 and 2, respectively. No significant differences were found for H3ac (p>0.9 and p>0.8, n = 4, for regions 1 and 2, respectively). (**D**) Analysis of Pol-II density in β-globin genes under low induction conditions. The density of Pol-II in the *mut* and *wt* β-globin genes was analyzed by ChIP as in (B) after induction with 20 µM CuSO_4_. The histogram shows average Pol-II signal relative to input and normalized to actin 5C from two independent experiments each quantified in duplicate (n = 4). Paired, two-tailed Student's t-test *p* values for comparisons of Pol-II density between *wt* and *mut* were 0.002 and 0.14 (not significant difference) for regions 1 and 2, respectively.

We subsequently analysed the levels of expression of the *wt* and *mut* β-globin genes. We first measured pre-mRNA and mRNA levels for *wt* and *mut* β-globin by RT-qPCR. The RT-qPCR signals were normalized to actin 5C mRNA. The level of *mut* β-globin pre-mRNA was approximately one third of the level of *wt* β-globin pre-mRNA, and the difference in mRNA levels was even greater, approximately 40 fold (Figure 4A). The mRNA is defined as transcript from which intron1 had been removed by splicing.

The S2 cells used in this study were stable transfectants generated with calcium chloride (see Materials and Methods S1). Cells transfected by this method usually carry multiple plasmid copies inserted in tandem in the genome. The differences in expression described above may have been due to a difference in the number of gene copies present in each cell line, and in order to determine whether this was the case we quantified by qPCR the genomic copy numbers of *wt* and *mut* β-globin genes, relative to actin 5C. We found that the S2 line that expresses the *mut* β-globin carries approximately twice as many globin gene copies as the strain that expresses the *wt* β-globin (data not shown). We concluded that the differences in transcript levels detected by RT-qPCR (Figure 4A) were not due to differences in gene copy number.

We performed chromatin immunoprecipitation (ChIP) with an antibody against the CTD, in order to quantify the density of Pol-II in the *wt* and *mut* β-globin genes. Quantification of the immunoprecipitated DNA was based on qPCR assays using primer pairs specific for either the upstream region of the β-globin gene, close to the promoter (*region 1* in Figure 4B), or the 3′ UTR (*region 2* in Figure 4B). The qPCR values obtained in each experiment were expressed relative to the input for that experiment, and normalized against actin 5C. The ChIP experiments showed that the density of Pol-II was higher near the promoter than in the downstream region of the gene. The same type of distribution has been observed in previous studies in both mammalian and insect cells [for example 23], and is explained by the fact that many Pol-II complexes fail to engage in efficient elongation. Moreover, the density of Pol-II was lower in the *mut* than in the *wt* β-globin gene, both near the promoter and at the 3′ UTR (Figure 4B). These observations show that the *mut* β-globin gene is less efficiently transcribed than the *wt* gene, and suggest that splicing influences transcription. The fact that the Pol-II density is different in the promoter-proximal region of the gene suggests that the splice sites influence transcription at the level of initiation.

Next we asked whether the presence of splice-site mutations had any influence on the chromatin state and we carried out ChIP experiments with antibodies against two different post-translational histone modifications that mark actively transcribed chromatin: trimethylation of lysine 4 in histone H3 (H3K4me3) and acetylation of histone H3 (H3ac). As shown in Figure 4C, the levels of H3K4me3 were lower in the *mut* than in the *wt* β-globin gene, whereas the global levels of H3ac did not show any significant difference. These observations suggest that the impaired transcription of the *mut* β-globin gene is accompanied by changes in chromatin structure that involve specific histone modifications.

The experiments reported above were carried out under conditions in which the expression of the β-globin genes was strongly induced with 400 µM CuSO_4_. We repeated the ChIP experiments using cells in which β-globin expression was weakly induced with 20 µM CuSO_4_. The density of Pol-II in the β-globin genes was lower under these low induction conditions in both the *wt* and *mut* genes (Figure S4), as expected. Interestingly, there was no difference in Pol-II density between *mut* and *wt* under low-induction conditions (Figure 4D).

In summary, these experiments allow us to conclude that the *mut* β-globin gene is expressed at a lower level than the *wt* β-globin gene, and that this difference is at least partly due to a reduced transcription initiation rate.

## Discussion

### The role of Rrp6 in the nuclear retention of unspliced mRNAs is evolutionarily conserved

The nuclear surveillance pathways can recognize at least three types of defect in gene expression: the presence of unspliced introns in the mRNA [32]–[34], inefficient polyadenylation [25], [35], and defects in RNP assembly or in the recruitment of export factors to the mRNP [36], [37]. Studies in budding yeast have revealed that the nuclear quality control mechanisms operate cotranscriptionally and at the nuclear envelope by targeting defective transcripts for degradation by the nuclear exosome [reviewed in 11]. The strong evolutionary conservation of the exosome implies that similar surveillance mechanisms exist in metazoans. This idea is supported by studies in mammalian cells that have shown that unprocessed RNAs are not efficiently released from the transcription site [24]. In *D. melanogaster*, and probably in all eukaryotes, the exosome associates with elongating transcription complexes and with nascent pre-mRNPs [22], [23]. However, it has not been established that the exosome is involved in the cotranscriptional surveillance of mRNA biogenesis in metazoans. We have studied the mechanisms of nuclear surveillance of mRNA biogenesis and the role of the nuclear exosome subunit Rrp6 in cotranscriptional surveillance using β-globin genes inserted into the genome of S2 cells. We have analyzed the expression of a *mut* β-globin gene that carries splice-site mutations that prevent the excision of intron 2. We have shown that the *mut* β-globin transcripts are properly spliced at intron 1 and are processed at the 3′ end. However, these *mut* transcripts retain intron 2 and are not efficiently released from the transcription site. Moreover, the *mut* β-globin genes are transcriptionally down-regulated and the levels of the *mut* β-globin transcripts are much lower than those of *wt* β-globin mRNAs expressed under the same conditions. Several conclusions can be drawn from these observations. Firstly, that insect cells also have a quality control checkpoint that acts at the transcription site to restrict the release of unprocessed mRNAs, as previously shown for yeast and mammals [reviewed in 9]. Secondly, that one splicing event is not sufficient for the release of the mRNA from the transcription site. And thirdly, that pre-mRNA processing defects feed back on transcription and down-regulate the synthesis of the defective transcripts (see below).

We have investigated the role of the nuclear exosome in cotranscriptional quality control and demonstrated that Rrp6 is required for the nuclear retention of unspliced transcripts. Knocking-down the exosome subunit Rrp6 improved the release of the *mut* transcripts from the transcription site. This observation reveals that the role of Rrp6 in the nuclear retention of defective transcripts is not restricted to yeast but is conserved in insects, and probably in all metazoans.

### The splicing-defective *mut* β-globin gene is transcriptionally downregulated

The analysis of the expression of *wt* and *mut* β-globin genes also revealed that the presence of splice-site mutations resulted in a marked reduction of transcription, assessed by the density of CTD in ChIP assays. Surveillance responses that feed back on transcription have been reported in both yeast and human cells [reviewed in 11]. In budding yeast, the Yra1 protein participates in mRNP assembly and mediates the association of mRNAs with export factors. Mutations in Yra1 cause defects in mRNP assembly and downregulate transcription through a mechanism that requires the Mlp1/2 proteins [36]. A similar process takes place in mammalian cells, in which pre-mRNA splicing and transcription are intimately related. A recent study using β-globin genes integrated into the genome of human HEK cells revealed that mutations of the 5′ splice site in the pre-mRNA inhibit transcription initiation by interfering with the assembly of preinititiation complexes [8]. The interdependence between splicing and transcription has been shown in the context of promoter-proximal introns, and it has been argued that the 5′ splice site needs to be close to the promoter for efficient enhancement of transcription [8], [38]. However, our results show that defects in the splicing of a downstream intron also reduce transcription initiation, in spite of the existence of a functional 5′ splice site close to the promoter. This suggests that the stimulating effect of the upstream splice site is not the only splicing-related mechanism that contributes to efficient transcription initiation. The β-globin gene is relatively short, and the mutated 5′ splice site of intron 2 is located approximately 500 bp from the transcription start site, a distance corresponding to approximately three nucleosomes in a chromatin context. It is conceivable that the 5′ splice site of intron 2 reaches back to the promoter and favors the assembly of preinitiation complexes, as Damgaard et al. proposed for intron 1 [8]. Alternatively, it is also possible that the nascent pre-mRNA recruits chromatin-modifying enzymes that establish a repressive environment not only at the promoter but along the entire gene. This mRNA-mediated response would discriminate between correct and defective mRNAs because correct mRNAs are rapidly processed and released from the transcription site, and thereby do not contribute to chromatin repression to the same extent as mutant transcripts. This type of mechanism is compatible with our ChIP results and with the observation by Damgaard et al. [8] that β-globin genes with mutated splice sites show reduced levels of active chromatin markers such as H3K9Ac and H3K4me3, and it is compatible with reports of the recruitment of chromatin modifiers by nascent RNAs [for example 39]–[41].

Interestingly, the transcriptional activity of the *mut* β-globin gene was lower than that of the *wt* only when gene expression was strongly induced: the densities of Pol-II were similar in *mut* and *wt* genes under low expression conditions. Defects in mRNP assembly and 3′ end processing are also rescued by lowered transcription rates in budding yeast [42]. Taken together, these findings suggest that lowering transcription is a general surveillance response that contributes to proper mRNP formation.

In summary, our results support the hypothesis that at least two surveillance responses operate cotranscriptionally in insect cells. One response reduces the transcript cleavage efficiency and results in inefficient release of defective mRNAs from the transcription site. The other response reduces the transcription of the genes that code for defective transcripts through a mechanism that affects the state of the chromatin. It remains to be investigated whether insect cells also have a surveillance mechanism associated with the nuclear envelope, as budding yeast has [34].

## Materials and Methods

### Plasmids

The *wt* and *mut* β-globin genes were subcloned from the plasmids pβΔRS [43] and pβDM [24] into the pMT/V5-HisB *Drosophila* expression vector (Invitrogen). For this purpose, the sequences that lay between the start and stop codons were amplified by PCR and ligated into the *KpnI XhoI* restriction sites of the pMT/V5-His B (Invitrogen). The ligation products were transformed into *E. coli* XL10-Gold. The resulting plasmids containing the *wt* and *mut* β-globin genes were referred to as pMT-βΔRS and pMT-DM, respectively. Both plasmids were sequenced to confirm the accuracy of the cloning.

### Culture and transfection of S2 cells

*D. melanogaster* S2 cells (Invitrogen) were cultured at 28°C according to the instructions of the *Drosophila Expression System* manual from Invitrogen. Stable cells were generated using the Calcium Phosphate Transfection Kit (Invitrogen). Stable transfectants were selected and maintained in the presence of 300 µg/ml hygromycin B. Expression of the β-globin genes was induced with CuSO_4_ during 24 hours. In some cases, the cells were treated with actinomycin D (5 to 7.5 µg/ml) for 15 minutes before fixation or adhesion to slides.

### Nuclear fractionation

*D. melanogaster* S2 cells from a 3.5 cm plate were harvested, washed with PBS, and lysed in 180 µl cold lysis buffer (50 mM Tris (pH 7.5), 150 mM NaCl, 1 mM MgCl_2_, 10% glycerol, 0.1% NP-40) for 5 min at 4°C (thermomixer, 1000 rpm). After centrifugation (10 min, 6000 g, 4°C), the supernatant was discarded and the pellet washed once more in lysis buffer. Total RNA of the pellet (nuclear fraction) was extracted using the RNAqueous kit (Ambion).

### RNA extraction and RT-PCR analysis

Total RNA from S2 cells was extracted using the RNAqueous kit (Ambion). Reverse transcription was performed with Superscript-III (Invitrogen) using random primers (Roche). The resulting cDNA was used as a template for PCR reactions using Taq polymerase (Fermentas). The conditions of the PCR reactions were optimized for each primer pair to ensure that the analysis was carried out within the linear range of the amplification.

### RT-qPCR

For real-time PCR (qPCR), reverse-transcribed material was amplified in 25 µl Power SYBR Green PCR Master Mix (Applied Biosystem) using the 7000 Sequence Detection System (Applied Biosystem) or in 20 µl KAPA SYBR Fast qPCR Master Mix (KAPA Biosystems) using the RotorGene (Qiagen). In most cases, actin 5C mRNA was used for normalization purposes. The sequences of all primers used can be found in the oligonucleotide list in the Materials and Methods S1.

### RNA interference in S2 cells

RNA interference was carried out essentially as described by Hase et al. [44]. DsRNAs against Rat1, Rrp6 and GFP were prepared by *in vitro* transcription from PCR products with T7 promoters on both ends of the amplimers, using the Megascript RNAi kit (Ambion). For FISH experiments, S2 cells were treated with 10 µg of dsRNA every 24 h, and the cells were analyzed after 5 days of dsRNA treatment. For RT-qPCR, the cells were treated with 20 µg dsRNA every second day and harvested after 4 days of dsRNA treatment.

### Immunofluorescence

S2 cells were allowed to adhere to poly-L-lysine-coated slides, fixed with 3.7% formaldehyde in PBS at room temperature (RT) for 10 min and permeabilized in 0.5% Triton X-100 in PBS at RT for 13 min. The cells were washed with PBS and stained using standard procedures. The slides were mounted in VectaShield Mounting Medium with DAPI (Vector Labs) and examined in a Zeiss Axioplan fluorescence microscope.

### FISH

S2 cells were allowed to adhere to poly-L-lysine-coated slides, fixed with 3.7% formaldehyde in PBS at RT for 10 min and permeabilized in 0.5% Triton X-100 in PBS at RT for 13 min. The cells were then washed with PBS, incubated in pre-hybridization solution, and hybridized at 37°C. The probe was the plasmid pβΔRS labeled with DIG-11-dUTP. The sites of hybridization were visualized using a FITC-conjugated antibody against DIG (Invitrogen) and an anti-FITC antibody coupled to Alexa488 (DakoCytomation). The preparations were finally mounted in VectaShield (Vector Labs). For quantitative analysis of the FISH results, the number of cells with a bright nuclear spot was counted in randomly selected fields. Ninety-eight cells were analyzed per slide, and the results from four slides from at least two different experiments were pooled together for each treatment (392 cells in total, n = 4). The average frequencies of cells showing a bright fluorescent spot were compared using Student's t-tests as indicated in the figure legends.

### Chromatin Immunoprecipitation (ChIP)

ChIP was performed essentially as described in [23] with a few modifications. For a detailed protocol see the Materials and Methods S1.

## Supporting Information

Figure S1 Nucleotide sequences of *wt* and *mut* β-globin transcripts. Total RNA was isolated from cells expressing either the *wt* or the *mut* β-globin genes. The RNA was reversed transcribed and the resulting cDNA was amplified by PCR using primers flanking intron 2. The amplified products were then analyzed by sequencing. The yellow box indicates exon 2, the green box is exon 3. Intron 2 of the *wt* transcript was spliced out, whereas it is still present in the *mut* transcript. The mutated splice sites are shown in red.
(0.72 MB DOC)

Figure S2 Rrp6 depletion analyzed by Western Blot. S2 cells expressing either the *wt* or *mut* β-globin gene were treated with either Rrp6- dsRNA or GFP-dsRNA as a control. The efficiency of the Rrp6 knockdown was monitored by Western blotting using an antibody against Rrp6. The asterisk indicates a cross-reactivity of the antibody to an unknown protein. Tubulin served as loading control. The level of depletion was above 95%.
(0.13 MB DOC)

Figure S3 Rat1 depletion analyzed by RT-qPCR. The expression of Rat1 was silenced by RNAi in S2 cells expressing either *mut* or *wt* β-globin. As a control, cells were treated in parallel with GFP-dsRNA. Total RNA was purified and reverse transcribed from dsRNA-treated cells, and the resulting cDNAs were analyzed by qPCR with primers specific for Rat1. The histogram shows average values normalized actin5C RNA levels from three independent experiments. The error bars represent standard deviations.
(0.11 MB DOC)

Figure S4 Analysis of Pol-II density in β-globin genes under different induction conditions. The expression of the β-globin genes was induced with either 20 or 400 µM CuSO4, and the density of Pol-II in the *mut* and *wt* β-globin genes was analyzed by ChIP using an anti-CTD antibody, as in Figure 4. The histogram shows average Pol-II signals relative to input and actin5C from one experiment with two qPCR runs and duplicates. The error bars represent standard deviations.
(0.11 MB DOC)

Materials and Methods S1 Detailed materials and methods.
(0.07 MB DOC)
