# Supplementary material for: Splice-Site Mutations Cause Rrp6-Mediated Nuclear Retention of the Unspliced RNAs and Transcriptional Down-Regulation of the Splicing-Defective Genes
Source: PLoS One. 2010 Jul 12;5(7):e11540. doi: 10.1371/journal.pone.0011540 (PMC2902512; doi:10.1371/journal.pone.0011540)
Supplement: Figure S1 — Nucleotide sequences of wt and mut β-globin transcripts. Total RNA was isolated from cells expressing either the wt or the mut β-globin genes. The RNA was reversed transcribed and the resulting cDNA was amplified by PCR using primers flanking intron 2. The amplified products were then analyzed by sequencing. The yellow box indicates exon 2, the green box is exon 3. Intron 2 of the wt transcript was spliced out, whereas it is still present in the mut transcript. The mutated splice sites are shown in red. (0.72 MB DOC) [file pone.0011540.s001.doc]

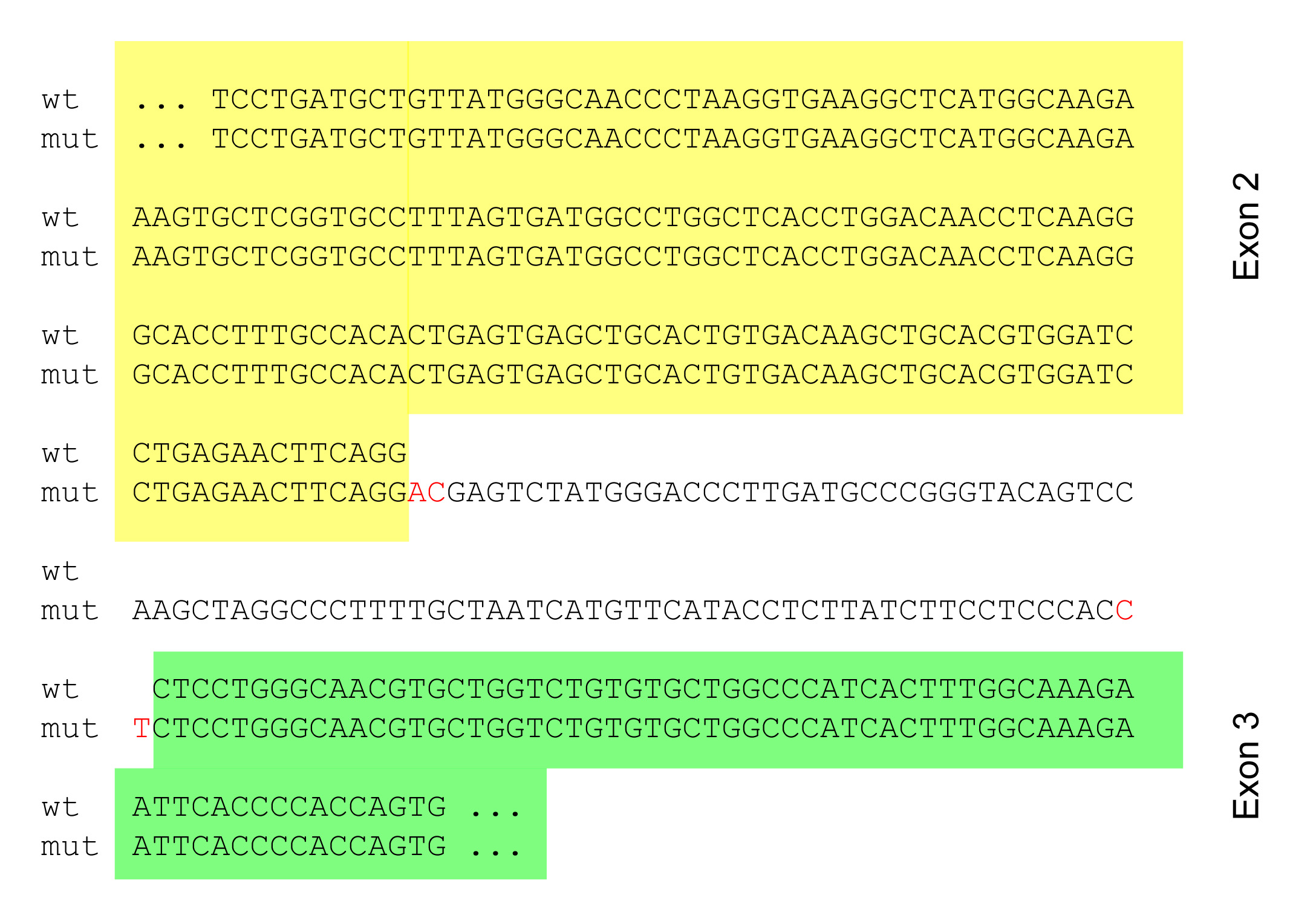


**Figure S1. Nucleotide sequences of *wt* and *mut* β-globin transcripts.**

Total RNA was isolated from cells expressing either the *wt* or the *mut* β-globin genes. The RNA was reversed transcribed and the resulting cDNA was amplified by PCR using primers flanking intron2. The amplified products were then analyzed by sequencing. The yellow box indicates exon 2, the green box is exon 3. Intron 2 of the *wt* transcript was spliced out, whereas it is still present in the *mut* transcript. The mutated splice sites are shown in red.
