# Supplementary material for: Splice-Site Mutations Cause Rrp6-Mediated Nuclear Retention of the Unspliced RNAs and Transcriptional Down-Regulation of the Splicing-Defective Genes
Source: PLoS One. 2010 Jul 12;5(7):e11540. doi: 10.1371/journal.pone.0011540 (PMC2902512; doi:10.1371/journal.pone.0011540)
Supplement: Figure S2 — Rrp6 depletion analyzed by Western Blot. S2 cells expressing either the wt or mut β-globin gene were treated with either Rrp6- dsRNA or GFP-dsRNA as a control. The efficiency of the Rrp6 knockdown was monitored by Western blotting using an antibody against Rrp6. The asterisk indicates a cross-reactivity of the antibody to an unknown protein. Tubulin served as loading control. The level of depletion was above 95%. (0.13 MB DOC) [file pone.0011540.s002.doc]

**
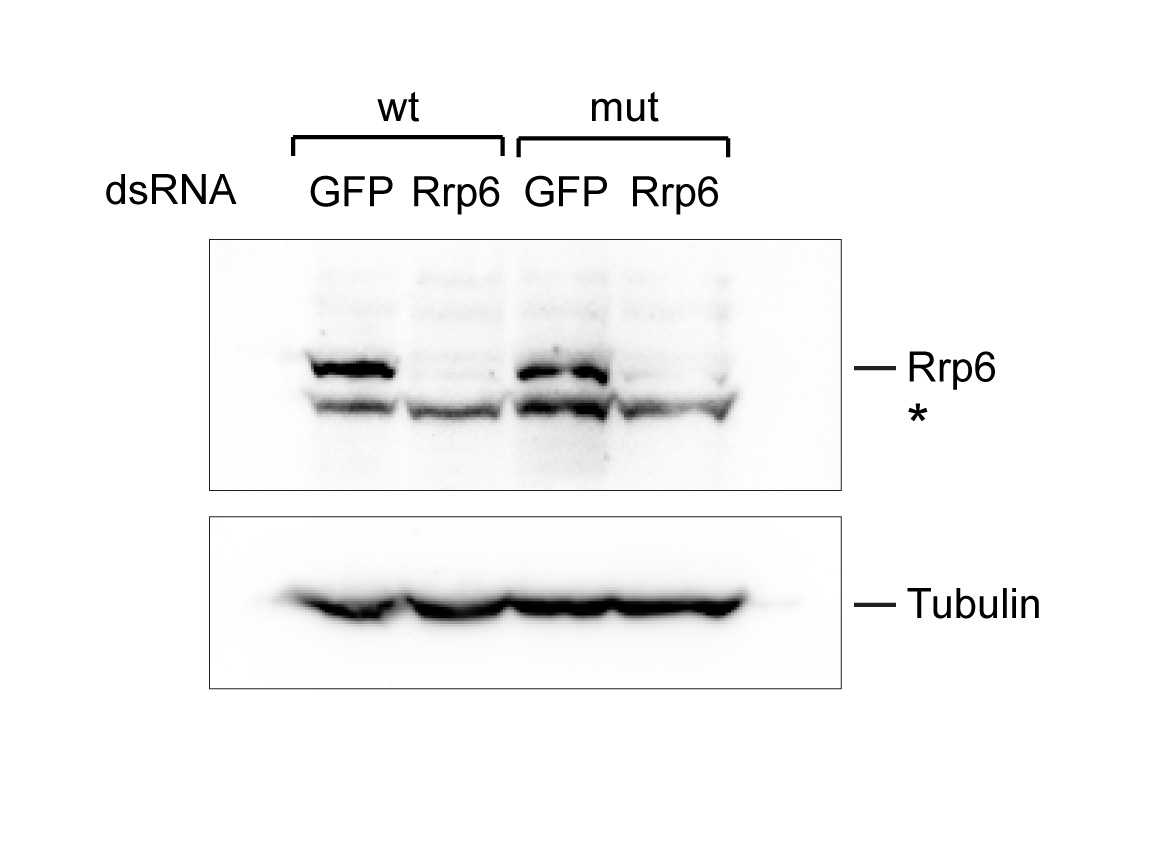
**

**Figure S2. Rrp6 depletion analyzed by Western Blot.**

S2 cells expressing either the *wt* or *mut* β-globin gene were treated with either Rrp6- dsRNA or GFP-dsRNA as a control. The efficiency of the Rrp6 knockdown was monitored by Western blotting using an antibody against Rrp6. The asterisk indicates a cross-reactivity of the antibody to an unknown protein. Tubulin served as loading control. The level of depletion was above 95%.
