# Supplementary material for: Splice-Site Mutations Cause Rrp6-Mediated Nuclear Retention of the Unspliced RNAs and Transcriptional Down-Regulation of the Splicing-Defective Genes
Source: PLoS One. 2010 Jul 12;5(7):e11540. doi: 10.1371/journal.pone.0011540 (PMC2902512; doi:10.1371/journal.pone.0011540)
Supplement: Figure S3 — Rat1 depletion analyzed by RT-qPCR. The expression of Rat1 was silenced by RNAi in S2 cells expressing either mut or wt β-globin. As a control, cells were treated in parallel with GFP-dsRNA. Total RNA was purified and reverse transcribed from dsRNA-treated cells, and the resulting cDNAs were analyzed by qPCR with primers specific for Rat1. The histogram shows average values normalized actin5C RNA levels from three independent experiments. The error bars represent standard deviations. (0.11 MB DOC) [file pone.0011540.s003.doc]

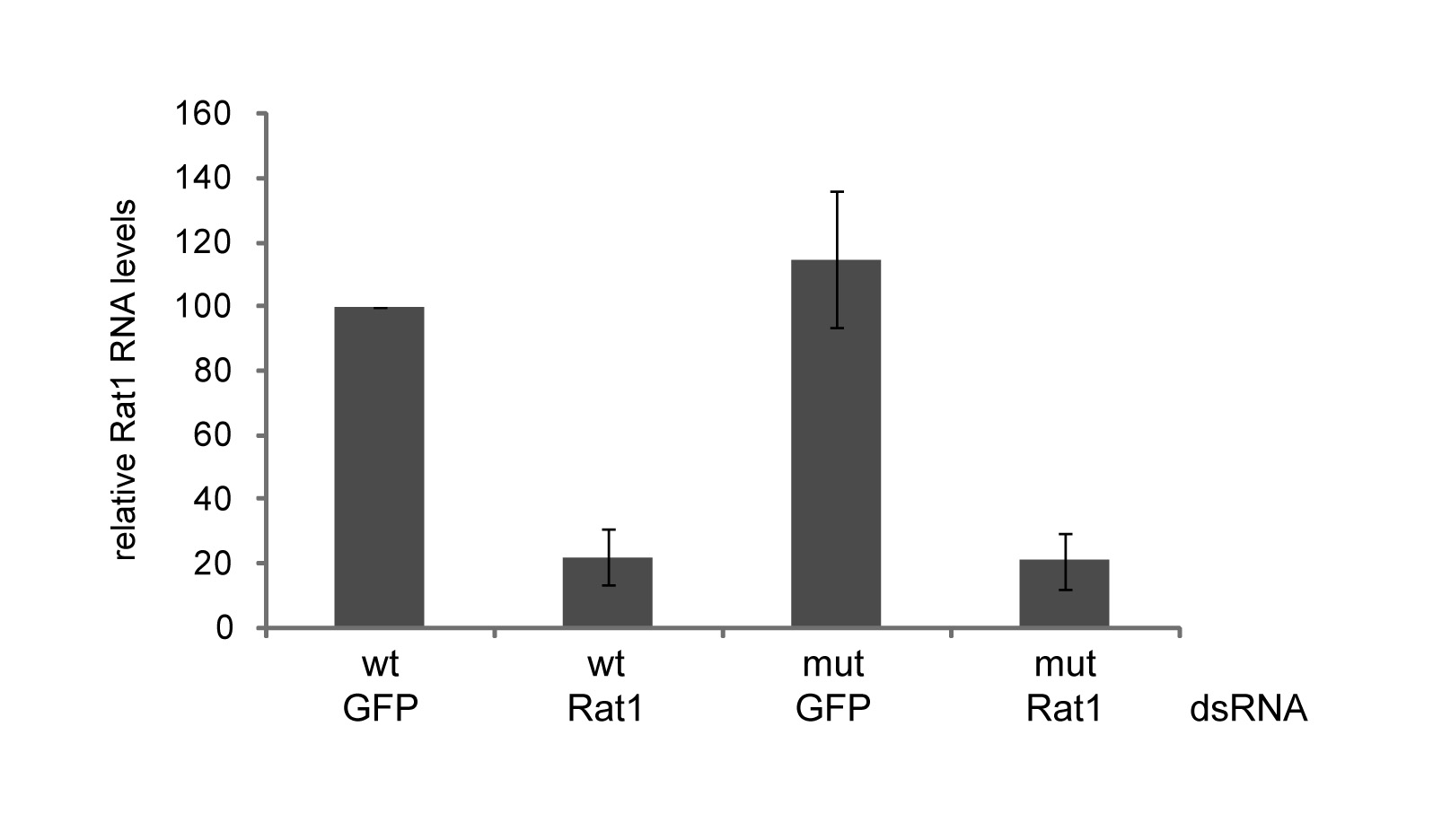


**Figure S3. Rat1 depletion analyzed by RT-qPCR.**

The expression of Rat1 was silenced by RNAi in S2 cells expressing either *mut* or *wt* β-globin. As a control, cells were treated in parallel with GFP-dsRNA. Total RNA was purified and reverse transcribed from dsRNA-treated cells, and the resulting cDNAs were analyzed by qPCR with primers specific for Rat1. The histogram shows average values normalized actin5C RNA levels from three independent experiments. The error bars represent standard deviations.
