# Supplementary material for: Splice-Site Mutations Cause Rrp6-Mediated Nuclear Retention of the Unspliced RNAs and Transcriptional Down-Regulation of the Splicing-Defective Genes
Source: PLoS One. 2010 Jul 12;5(7):e11540. doi: 10.1371/journal.pone.0011540 (PMC2902512; doi:10.1371/journal.pone.0011540)
Supplement: Figure S4 — Analysis of Pol-II density in β-globin genes under different induction conditions. The expression of the β-globin genes was induced with either 20 or 400 µM CuSO4, and the density of Pol-II in the mut and wt β-globin genes was analyzed by ChIP using an anti-CTD antibody, as in Figure 4. The histogram shows average Pol-II signals relative to input and actin5C from one experiment with two qPCR runs and duplicates. The error bars represent standard deviations. (0.11 MB DOC) [file pone.0011540.s004.doc]

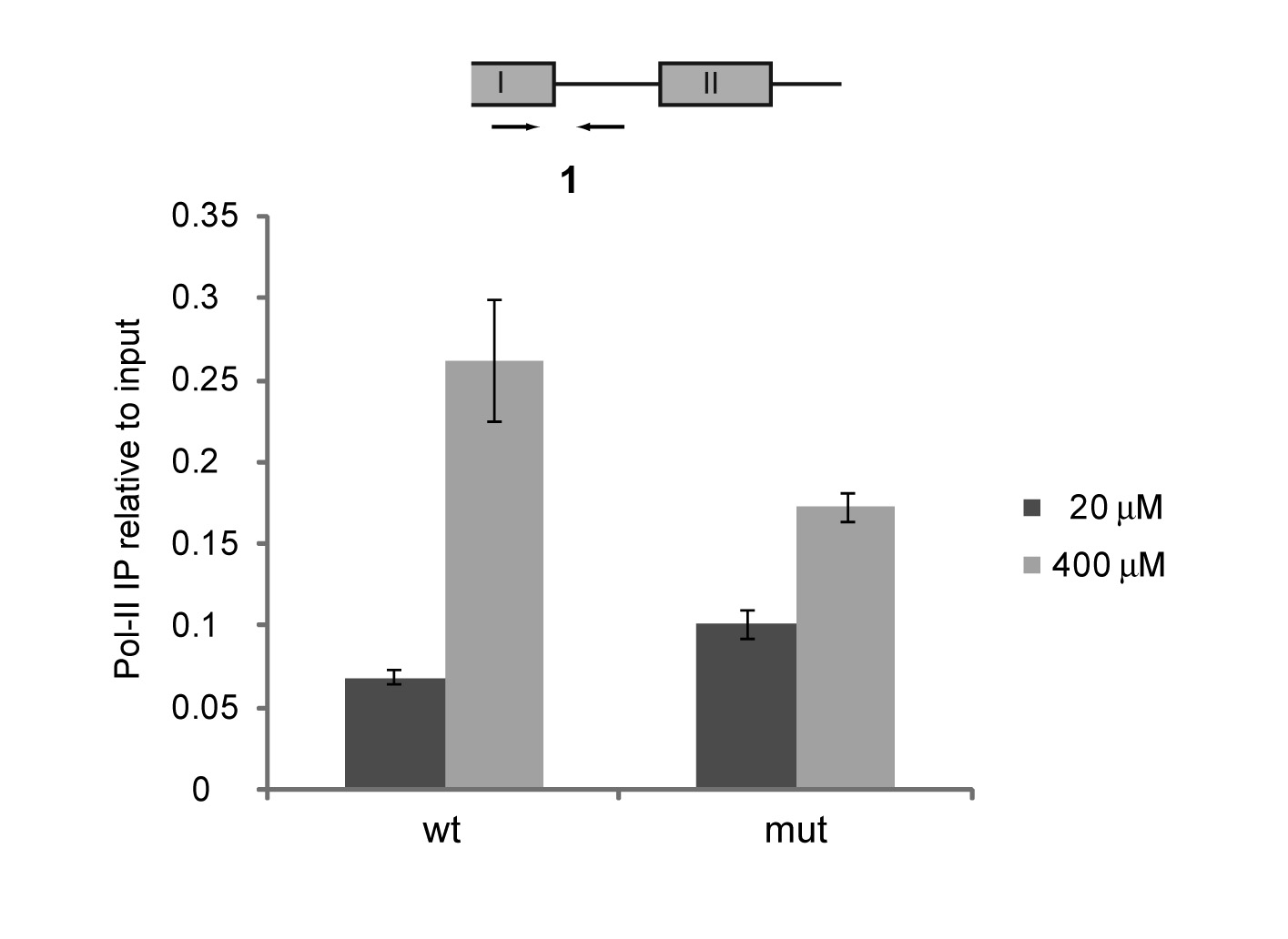


**Figure S4. Analysis of Pol-II density in β-globin genes under different induction conditions.**

The expression of the β-globin genes was induced with either 20 or 400 µM CuSO_4_, and the density of Pol-II in the *mut* and *wt* β-globin genes was analyzed by ChIP using an anti-CTD antibody, as in Figure 4. The histogram shows average Pol-II signals relative to input and actin5C from one experiment with two qPCR runs and duplicates. The error bars represent standard deviations.
