# Supplementary material for: Splice-Site Mutations Cause Rrp6-Mediated Nuclear Retention of the Unspliced RNAs and Transcriptional Down-Regulation of the Splicing-Defective Genes
Source: PLoS One. 2010 Jul 12;5(7):e11540. doi: 10.1371/journal.pone.0011540 (PMC2902512; doi:10.1371/journal.pone.0011540)
Supplement: Materials and Methods S1 — Detailed materials and methods. (0.07 MB DOC) [file pone.0011540.s005.doc]

***Supporting Materials and Methods***

***Plasmids***

The *wt* and *mut* ß-globin genes were subcloned from the plasmids pβΔRS [1] and pβDM [2] into the pMT/V5-HisB Drosophila expression vector (Invitrogen). For this purpose, the sequences comprised between the start and stop codons were amplified by PCR with Herculase Enhanced DNA Polymerase (Stratagene) using the above mentioned plasmids as templates. The forward primer (*forward-Kpn*) contained a *Kpn*I restriction site incorporated upstreams of the translation initiation codon. The reverse primer (*reverse-Xho*) contained a *Xho*I restriction site. The resulting PCR product was digested with *Kpn*I (Invitrogen) and *Xho*I (NEB) and ligated with the digested vector pMT/V5-His B (Invitrogen). The ligation products were transformed into *E. coli* XL10-Gold cells using the calcium chloride method [3]. The resulting plasmids containing the *wt* and *mut* ß-globin genes were referred to as pMT-βΔRS and pMT-DM, respectively. Both plasmids were sequenced to confirm the accuracy of the cloning.

***Culture and transfection of S2 cells***

*D. melanogaster* S2 cells were cultured at 28°C according to the instructions of the *Drosophila Expression System* manual from Invitrogen. Stable cells were generated by cotransfecting S2 cells with pCoHygro (Invitrogen) and either pMT-βΔRS or pMT-DM using the Calcium Phosphate Transfection Kit (Invitrogen). Stable transfectants were selected in the presence of 300 µg/ml hygromycin B following the procedure recommended by Invitrogen. Expression of the ß-globin genes was induced with 400 μM CuSO_4_ during 24 hours. In some cases, the cells were treated with actinomycin D (5 to 7.5 µg/ml) for 15 minutes before fixation.

### ***SDS-PAGE and Western*** ***blotting***

### Proteins were separated by SDS-PAGE using the Mini-Protean II system (BioRad) and transferred to polyvinylidenefluoride (PVDF) membranes (Millipore) in Tris-glycine buffer with 0.02% SDS and 4 M urea using a semi-dry electrophoretic transfer cell (BioRad). The membranes were probed with antibodies following standard procedures.

## RNA extraction and RT-PCR analysis

## Total RNA from S2 cells was extracted using the RNAqueous kit (Ambion). Reverse transcription was performed with Superscript-III (Invitrogen) using random primers (Roche). The resulting cDNA was used as a template for PCR reactions using Taq polymerase (Fermentas). All used primers can be found in the primer list below. The conditions of the PCR reaction were optimized for each primer pair to ensure that the analysis was carried out within the linear range of the amplification.

***qPCR and qRT-PCR***

For real-time PCR (qPCR), reverse transcribed material was amplified in 25 µl Power SYBR Green PCR Master Mix (Applied Biosystem) using the 7000 Sequence Detection System (Applied Biosystem) or in 20 µl KAPA SYBR Fast qPCR Master Mix (KAPA Biosystems) using RotorGene (Qiagen). All primers used for qPCR are specified in the primer list below.

***RNA interference in S2 cells***

RNA interference was carried out essentially as described by Hase et al. [4]. DsRNAs against Rrp6, Rat1 and GFP were prepared by *in vitro* transcription from PCR products with T7 promoters on both ends of the amplimers, using the Megascript RNAi kit (Ambion). For FISH experiments, S2 cells were treated with 10 µg of dsRNA every 24 h, and the cells were analyzed after 5 days of dsRNA treatment. For qRT-PCR, the cells were treated with 20 µg dsRNA every second day and harvested after 4 days of dsRNA treatment.

***Immunofluorescence***

S2 cells were let to adhere to poly-L-lysine-coated slides, fixed with 3.7 % formaldehyd in PBS at RT for 10 min and permeabilized in 0.5% Triton X-100 in PBS at RT for 13 min. The cells were washed with PBS, blocked with 3% BSA in PBS for 30 min and incubated with an anti-V5 antibody (Invitrogen) for 1 hour. The antibody binding was visualized using a secondary antibody conjugated to Texas-Red (Jackson ImmunoResearch). The slides were mounted in VectaShield Mounting Medium with DAPI (Vector Labs) and examined in a Zeiss Axioplan fluorescence microscope.

## FISH

S2 cells were let to adhere to poly-L-lysine-coated slides, fixed with 3.7 % formaldehyd in PBS at RT for 10 min and permeabilize in 0.5% Triton X-100 in PBS at RT for 13 min. The cells were then washed with PBS, incubated in pre-hybridization solution (50% deionized formamide in 2xSSC containing 50 mM sodium phosphate pH 7.0) at 37°C for 10 min, and hybridized in the presence of 5µl heat denatured DIG-labeled probe (10-20 ng/µl) ON at 37°C. The probe was the plasmid pβΔRS labeled with DIG-11-dUTP by nick-translation using the DIG-nick-translation kit (Roche). Post-hybridization washes were done in 50 % formamide, 2xSSC at 45°C three times for 5 min each, 2xSSC at 45°C three times for 5 min each, and 4xSSC containing 0.1 % Tween20 for 5 minutes at 37 °C. The sites of hybridization were visualized using a FITC-conjugated antibody against DIG (Invitrogen) and an anti-FITC antibody coupled to Alexa488 (DakoCytomation). The preparations were finally mounted in VectaShield (Vector Labs). For the quantitative analysis of the FISH results, the number of cells with a bright nuclear spot was counted in randomly selected fields. 98 cells were analyzed per slide, and the results from four slides from at least two different experiments were pooled together for each treatment (392 cells in total). The average frequencies of cells showing a bright fluorescent spot were compared using a Student’s t-test.

### ***Image acquisition***

The preparations were examined and images were taken with a Zeiss Axioplan microscope equiped with PlanApochromat objectives 40x/1.0 oil and 63x/1.4 oil, using immersion oil Immersol™ 518F (Zeiss). Photoshop and Illustrator software (Adobe) was used for image processing and elaboration of composite figures.

***Chromatin immunoprecipitation***

The cells were fixed at room temperature for 10 min by the addition of a fixing solution containing formaldehyde (final concentration 2%). After incubation with 0.1 mM glycine for 10 min, the cells were spun down at 500 g for 5 min. The pellet was resuspended in cold buffer 1 (50 mM Hepes, pH 7.6, 140 mM NaCl, 1 mM EDTA, 10% glycerol, 0.5% NP-40, 0.25% Triton-X, Complete protease inhibitors (Roche)) and incubated 10 min at 4°C. The cells were spun down again as above. After centrifugation, the cells were resuspended in buffer 2 (10 mM Tris, pH 8, 200 mM NaCl, 1 mM EDTA, 0.5 mM EGTA, protease inhibitors) and incubated at room temperature for 10 min. The samples were centrifuged and the pellet resuspended in buffer 3 (10 mM Tris, pH 8, 1 mM EDTA, 0.5 mM EGTA, protease inhibitors). The obtained chromatin was sheared by sonication to give a DNA size of 250-900 bp. After centrifugation at 16 000 g for 30 min at 4°C, the samples were pre-cleared with Sepharose A/G slurry (50:50) for 2 hours on a rotating wheel. Immunoprecipitation was performed over-night at 4°C with primary antibody in the presence of 0.1% deoxycholic acid and 1% triton. Blocked A/G slurry was added and incubation was prolonged for one hour. After incubation, the samples were spun down and washed 5 times 10 minutes with RIPA buffer (50 mM Hepes, pH 7.6, 500 mM NaCl, 1 mM EDTA, 1 % NP-40, 0.8 % deoxycholic acid). The last washing step was performed with 50 mM Tris, pH 8 and 2 mM EDTA. The pellet was then incubated in TE-Buffer including SDS, RNAse A and proteinase K for 3 hours at 55°C and over-night at 65°C. DNA was extracted with phenol:chloroform and precipitated with ethanol. The amounts of immunoprecipitated DNA were quantified by qPCR. The results of the Pol-II ChIP were expressed relative to input and the results from the histone modifications were expressed relative to histone H3. In all cases, the signals were also normalized to the amount of actin 5C detected in each sample.

The antibodies used for ChIP were from Abcam (ab5408 against Pol-II, ab1791 against histone H3, ab12209 against H3K4me3) and from Millipore (06-599 against H3 acetylation).

**References**

1. Antoniou M, Geraghty F, Hurst J, Grosveld F (1998). Efficient 3’-end formation of human beta-globin mRNA in vivo requires sequences within the last intron but occurs independently of the splicing reaction. Nucleic Acids Res 26: 721-729.

2. Custodio N, Carmo-Fonseca M, Geraghty F, Pereira HS, Grosveld F et al. (1999) Inefficient processing impairs release of RNA from the site of transcription. EMBO J 18: 2855-2866.

3. Sambrook J, Fritsch EF, Maniatis T (1989) Molecular Cloning: A Laboratory Manual, Cold Spring Harbor, NY: Cold Spring Harbor Laboratory Press.

4. Hase ME, Yalamanchili P, Visa N (2006) The Drosophila heterogeneous nuclear ribonucleoprotein M protein, HRP59, regulates alternative splicing and controls the production of its own mRNA. J Biol Chem 281: 39135-39141.

**Sequences of the primers used in this study**

| **Name** | **Sequence** | **used in/for** |
| --- | --- | --- |
| Forward-Kpn | 5’-ttcggtaccatggtgcacctgactcctga-3’ | Cloning into pMT |
| Reverse-Xho | 5’-acttctcgagtgatacttgtgggccaggg-3’ | Cloning into pMT |
| ß-globin exon2 for | 5’-ttggacccagaggttctttg-3’ | Figure 1D |
| ß-globin exon3 rev | 5’-cactggtggggtgaattctt-3’ | Figure 1D |
| ß-globin exon1 for | 5’-acgtggatgaagttggtggt-3‘ | Figure 3A, 4A (pre-mRNA), Figure 4B-D, S4 (region1) |
| ß-globin intron1 rev | 5’-gcccagtttctattggtctcc-3’ | Figure 3A, 4A, (pre-mRNA), Figure 4B-D, S4 (region1) |
| ß-globin exon1/2 for | 5’-cctgggcaggctgctgg-3’ | Figure 3A, 4A (mRNA) |
| ß-globin exon2 rev | 5’-ctttcttgccatgagccttc-3’ | Figure 3A, 4A (mRNA) |
| ß-globin 3’fragment for | 5’-gggaggtgtgggaggtttt- 3’ | Figure 3A (3’fragment) |
| ß-globin 3’fragment rev | 5’-gcctgcaggtcgactgat-3’ | Figure 3A (3’fragment) |
| ß-globin 3’UTR for | 5’-gtgaaatttgtgatgctattgct-3’ | Figure 3B, 3C (poly(A)) |
| ß-globin polyA rev | 5’-tttttttttttgttgttgttaacttg-3’ | Figure 3B, 3C (poly(A)) |
| ß-globin polyA control rev | 5’-tgttgttgttaacttg-3’ | Figure 3B (poly(A) control) |
| ß-globin exon3 for | 5’-ggctgcctatcagaaagtgg-3’ | Figure 3C (normalization),  Figure 4B-D (region2) |
| ß-globin 3’UTR rev | 5’-agaccgaggagagggttagg-3’ | Figure 3C (normalization),  Figure 4B-D (region2) |
| Actin 5C for | 5’-gcacacccacaagcttacaca-3’ | Figure 3A, 4A-D, S3, S4 (normalization) |
| Actin 5C rev | 5’-ttgcgctttgggaaatatcttc-3’ | Figure 3A, 4A-D, S3, S4 (normalization) |
| Rat1 for | 5’-tcgaccgtctgttcggtatt-3’ | Figure S3 (RNAi efficiency) |
| Rat1 rev | 5’-attcatcttggctcgtggag-3’ | Figure S3 (RNAi efficiency) |
| Rrp6 for | 5’-cttcgaaaagcagtccaagc-3’ | Figure 2C (RNAi efficiency) |
| Rrp6 rev | 5’-cagctgcaccatcagaggta-3’ | Figure 2C (RNAi efficiency) |
| Fur2 for | 5’-tggagcgcagtcgaggtt-3’ | Figure 2C (control) |
| Fur2 rev | 5’-gaaagagaacgcatgcttacca-3’ | Figure 2C (control) |
| Tctp for | 5’-caagccaatcaccatgaagatc-3’ | Figure 2C (control) |
| Tctp rev | 5’-cacatcatccaccagcttcatc-3’ | Figure 2C (control) |
